# Clinical and Molecular Spectrum of Liposarcoma

**DOI:** 10.1200/JCO.2017.74.9598

**Published:** 2017-12-08

**Authors:** Alex Thomas John Lee, Khin Thway, Paul H. Huang, Robin Lewis Jones

**Affiliations:** Alex Thomas John Lee, Khin Thway, and Robin Lewis Jones, The Royal Marsden NHS Foundation Trust; Alex Thomas John Lee, Paul H. Huang, and Robin Lewis Jones, The Institute of Cancer Research, London, UK.

## Abstract

Liposarcomas are rare malignant tumors of adipocytic differentiation. The classification of liposarcomas into four principal subtypes reflects the distinct clinical behavior, treatment sensitivity, and underlying biology encompassed by these diseases. Increasingly, clinical management decisions and the development of investigational therapeutics are informed by an improved understanding of subtype-specific molecular pathology. Well-differentiated liposarcoma is the most common subtype and is associated with indolent behavior, local recurrence, and insensitivity to radiotherapy and chemotherapy. Dedifferentiated liposarcoma represents focal progression of well-differentiated disease into a more aggressive, metastasizing, and fatal malignancy. Both of these subtypes are characterized by recurrent amplifications within chromosome 12, resulting in the overexpression of disease-driving genes that have been the focus of therapeutic targeting. Myxoid liposarcoma is characterized by a pathognomonic chromosomal translocation that results in an oncogenic fusion protein, whereas pleomorphic liposarcoma is a karyotypically complex and especially poor-prognosis subtype that accounts for less than 10% of liposarcoma diagnoses. A range of novel pharmaceutical agents that aim to target liposarcoma-specific biology are under active investigation and offer hope of adding to the limited available treatment options for recurrent or inoperable disease.

## INTRODUCTION

Liposarcomas (LPSs) are malignant tumors of adipocytic differentiation. They are among the more common soft tissue sarcoma (STS) subtypes, accounting for approximately 15% to 20% of all STSs.^1^ This disease is classified into four principal subtypes: well-differentiated LPS (WDLPS; also known as atypical lipomatous tumor), dedifferentiated LPS (DDLPS), myxoid LPS (MLPS), and pleomorphic LPS (PLPS; Table 1).^2^ An improved appreciation of the contrasting clinical behaviors of these subtypes, coupled with a growing understanding of their underlying molecular pathology, has led to increasingly subtype-tailored management and the development of novel systemic therapies. In this article, we review the key clinical, pathologic, and molecular characteristics of the LPS subtypes, summarize current management, and provide an overview of ongoing investigation of new therapeutic strategies.

**Table 1.**
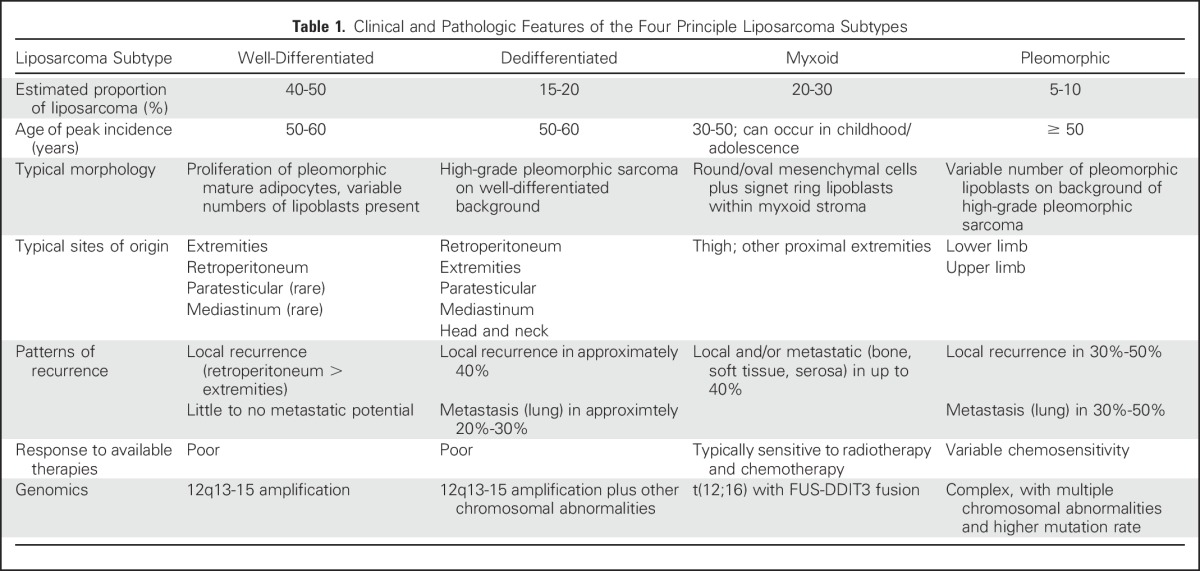
Clinical and Pathologic Features of the Four Principle Liposarcoma Subtypes

## LPS SUBTYPES

### WDLPS and DDLPS

**Fig 1.**
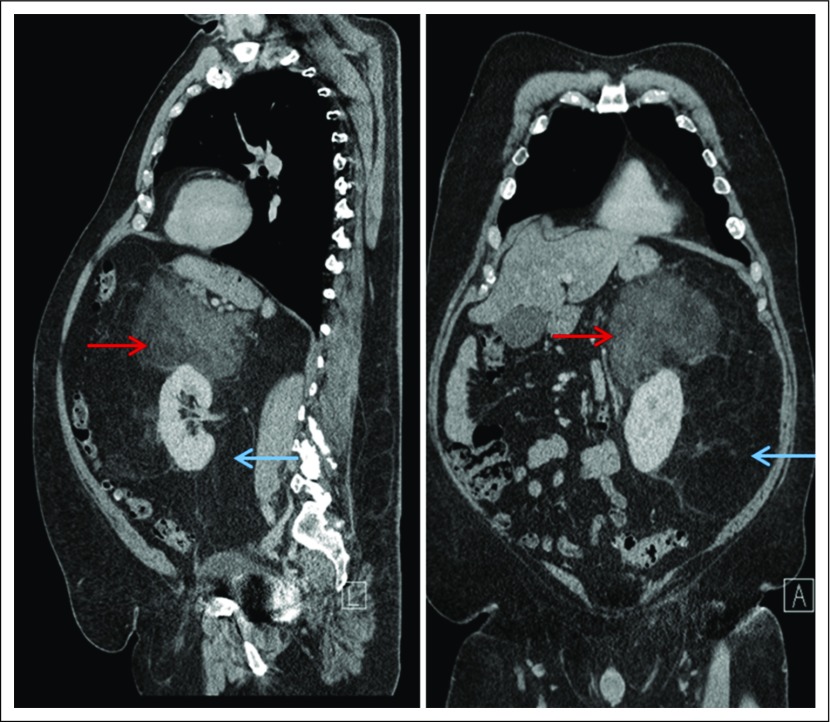
Radiologic appearance of retroperitoneal well-differentiated/dedifferentiated liposarcoma. Sagittal and coronal precontrast computed tomography images of a large liposarcoma expanding the retroperitoneum and encasing and displacing the left kidney. The blue arrow denotes a well-differentiated tumor, which shares a similar appearance to normal fat, extending inferiorly to the pelvic brim. The red arrow denotes a complex, ill-defined solid component of mixed attenuation, representing an area of dedifferentiated disease superior to left kidney.

WDLPS and DDLPS together account for the majority of LPSs and often coexist (WD/DDLPS). WDLPS can present as slowly growing masses in the retroperitoneum and proximal extremities. Distinguishing between peripheral WDLPS and much more commonly encountered benign adipocytic neoplasms may be challenging—lesions that are > 5cm in diameter, rapidly growing, and/or deep to superficial fascia warrant specialist evaluation. WDLPSs have no metastatic potential and are associated with an excellent outcome when complete excision is achieved. Local recurrence is more common when WDLPS arises in the retroperitoneum, mediastinum, or paratesticular region^3^ and is a cause of morbidity and mortality, as is the emergence of dedifferentiated disease. DDLPS is a high-grade and aggressive disease, arising most commonly within the retroperitoneum, and is associated with high rates of local and metastatic recurrence and disease-specific mortality that is six-fold that of WDLPS.^4^ Both WDLPS and DDLPS are typically radioinsensitive and chemoinsensitive.^5^ Shared morphologic and molecular features indicate that DDLPS occurs as a focal outgrowth within precursor WDLPS lesions, with 90% of DDLPS found within a primary WDLPS lesion and 10% within areas of locally recurrent WDLPS^6^ (Fig 1).

**Fig 2.**
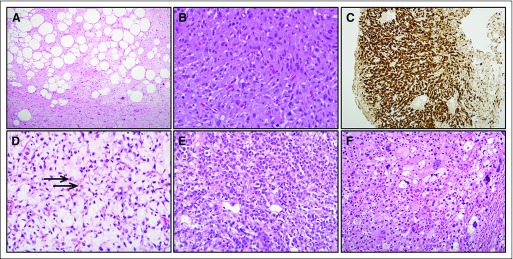
Histologic appearance of liposarcoma subtypes. (A) Hematoxylin and eosin stain of well-differentiated liposarcoma (× 40). Tumor is composed of mature adipocytes in normal adipose tissue prominently intersected by sparsely cellular fibrous septa containing atypical, enlarged spindle cells with hyperchromatic nuclei. (B) Dedifferentiated liposarcoma (× 200). Hematoxylin and eosin stain illustrates typical appearance of dedifferentiated component as a high-grade spindle or pleomorphic sarcoma, with sheets of moderately atypical spindle cells with scattered mitotic figures and no apparent adipocytic differentiation. (C) Immunohistochemistry staining of dedifferentiated liposarcoma (× 40) shows diffuse and strong expression of CDK4, frequently coamplified with MDM2 in well-differentiated and dedifferentiated liposarcoma. (D) Myxoid liposarcoma (× 200). Relatively bland and uniform appearance, with small ovoid or spindle cells dispersed in prominent myxoid stroma alongside plexiform network of curvilinear, thin-walled blood vessels. In many areas, small lipoblasts with nuclear indentation and vacuolated cytoplasm are identifiable (arrows). (E) Round cell variant of myxoid liposarcoma (× 40). Markedly cellular distribution of round and ovoid cells containing rounded, hyperchromatic nuclei and minimal, largely amphophilic cytoplasm. As in this example, the absence of discernible myxoid stroma can lead to round cell myxoid liposarcoma being mistaken for other round cell neoplasms such as Ewing sarcoma. (F) Pleomorphic liposarcoma (× 200). Large, atypical multivacuolated lipoblasts with indented hyperchromatic nuclei are dispersed in a background of atypical spindle cells.

Histologically, WDLPS appears as a proliferation of mature and variably pleomorphic adipocytes intersected by fibrous septa and containing single, enlarged, hyperchromatic nuclei (Fig 2A). DDLPS is characterized by more highly cellular areas of high-grade undifferentiated sarcoma typically transitioning abruptly within a background of WDLPS (Fig 2B). Supernumerary ring or giant rod chromosomes are found in both WDLPS and DDLPS. These consist of amplified segments of 12q13-15 that contain a number of cancer-related genes implicated in tumorigenesis.^7^ The most extensively studied of these are *MDM2*, an E3 ubiquitin protein ligase that acts as a key negative regulator of p53 and is amplified in nearly 100% of patients, and cyclin-dependent kinase 4 (*CDK4*), a key regulator of the G1/S cell cycle checkpoint that is coamplified with *MDM2* in over 90% of patients^8^ (Fig 2C). Other genes commonly coamplified within the 12q13-15 amplicon include *HMG2A*, encoding an architectural transcription factor shown to be capable of cellular transformation, and *TSPAN31*, a gene of currently unknown function that has been shown to be amplified in multiple STS subtypes.^9,10^ *YEATS4* and *CPM* are genes commonly coamplified within 12q13-15 that have been implicated in dedifferentiation.^11,12^ YEATS4 is a putative transcription factor required for physiologic suppression of p53 function and is implicated in oncogenesis across a number of cancers. In a large-scale genomic screening study of DDLPS cells, YEATS4 knockdown conferred greater antiproliferative effect than loss of MDM2 expression.^11^ *CPM* encodes carboxypeptidase M, a proteolytic enzyme with roles that include cleavage activation of growth factors, including epidermal growth factor. In a study of 12q-amplified LPS cell lines and xenografts, *CPM* knockout resulted in inhibition of growth, migration, and invasion, in association with downregulation of MAPK and PI3K pathway signaling.^12^

Although the somatic mutation rate has been shown to be low in WDLPS and DDLPS, the accumulation of additional chromosomal abnormalities seems to be central to the development of DDLPS.^11,12^ Recurrent amplifications of 1p32 and 6q23 are found exclusively in DDLPS and are associated with a worse prognosis.^13^ Overexpression of *ASK1* (found in 6q23) and *JUN* (1p32) has been implicated in dedifferentiation through mechanisms that involve inhibition of peroxisome proliferator-activated receptor gamma, (PPARγ), a key mediator of adipocyte differentiation.^14,15^ LPS genomic profiling studies have also identified chromosomal deletions of tumor suppressor genes such as *RB1* (13q14.2), *ATM,* and *CHEK1* (both 11q22-24), and *RUNX3* and *ARID1A* (both 1p36), that variably demonstrate association with reduced adipocytic differentiation, increased genomic instability, and worse clinical outcome.^11,12^ A potential role for receptor tyrosine kinase–mediated oncogenicity is suggested by the identification in DDLPS cell lines and clinical samples of chromosomal amplicons that contain *DDR2*, *ERBB3*, *FGFR1*, and *ROS1*.^16^

### Myxoid LPS

**Fig 3.**
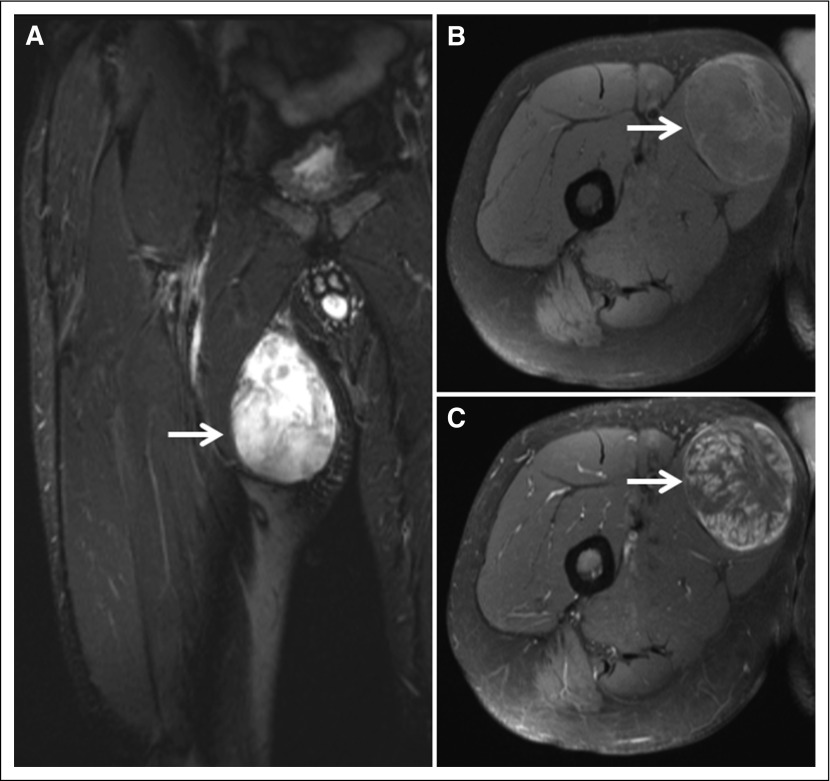
Radiologic appearance of myxoid liposarcoma of the proximal thigh. (A) Coronal T2-weighted magnetic resonance image of 80-mm tumor (arrow) with characteristic high signal intensity. T1-weighted fat-suppressed axial magnetic resonance images of (B) pregadolinium and (C) postgadolinium contrast show avid and heterogeneous enhancement of tumor (arrow).

MLPS accounts for approximately 30% of LPSs and is clinically and pathologically distinct from WD/DDLPS.^2^ Over 90% of MLPSs contain a pathognomonic t(12;16)(q13;p11) translocation that results in expression of the FUS-DDIT3 fusion protein, whereas a smaller proportion carries *EWSR1-DDIT3* gene fusions.^17^ Microscopically, MLPS has small, round-to-oval, nonadipocytic mesenchymal tumor cells alongside a variable number of immature lipoblasts on a background of prominent myxoid stroma (Fig 2D). Round cell LPS is now recognized as a high-grade, more cellular variant of MLPS that is associated with worse outcomes^2,18^ (Fig 2E). MLPS typically develops in the proximal extremities, with two thirds of cases originating in the thigh (Fig 3). Local recurrence and metastasis to atypical sites such as bone, retroperitoneum, serosal surfaces, and/or contralateral limb are commonly encountered. In addition to an increased round cell component, higher histologic grade, multifocality, and overexpression of p53 have been associated with an adverse prognosis.^18,19^ MLPSs are markedly more chemosensitive and radiosensitive than WD/DDLPSs.^5^

The FUS-DDIT3 fusion protein is believed to confer tumorigenicity in MLPS through dysregulation of adipocyte differentiation, leading to unchecked proliferation of immature lipoblasts that are incapable of differentiating.^20^ FUS is an FET family protein involved in regulation of transcription and RNA splicing, whereas DDIT3 is a member of the C/EBP transcription factor family that plays a role in adipocyte differentiation.^21^ Variant translocations, such as t(12;22)(q13;q12), resulting in fusion of *EWS*, another FET family member, to *DDIT3*, are likely to have similar mechanisms of oncogenesis.

Gene expression studies of MLPS have identified recurrent upregulation of *MET*, *RET*, and *PIK3CA,* indicating that these oncogenes may be under downstream transcriptional control by fusion proteins.^22^ Activating mutations or amplification of *PIK3CA* are seen in approximately 15% of MLPS, whereas *PTEN* deletion has also been described.^11,23^

### Pleomorphic LPS

PLPS is a rare and clinically aggressive LPS subtype. Typically arising in the limbs or, less commonly, the trunk or retroperitoneum, PLPS histologically appears as a high-grade undifferentiated sarcoma without recognizable lineage and contains a variable number of pleomorphic lipoblasts (Fig 2F). Distant metastases develop in 30% to 50% of patients, typically involving the lungs, and are generally unresponsive to chemotherapy or radiotherapy.^24,25^ Tumor-associated mortality occurs in up to 50% of patients.^26^

Current understanding of the molecular pathology of PLPS is limited. Characteristically, PLPSs have complex karyotypes consisting of multiple chromosomal losses and gains, indicating pathogenesis driven by complex and variable molecular events.^21^ Deletion of 13q14.2-5 (containing *RB1*) has been described in up to 50% of patients.^11,27^ Mutation or loss of *TP53* is also seen, in contrast to other forms of LPS where *TP53* alteration is uncommon.^11^ Loss of the tumor suppressor gene *NF1* is exhibited in a proportion of patients, whereas epigenetic silencing of the p53 target gene *p14ARF* has been implicated as playing a role in tumorigenesis.^12,28^

## CURRENT MANAGEMENT

### Localized Disease

Wide local excision with clear surgical margins is the core curative treatment in localized LPS. The optimal extent of resection for retroperitoneal LPS is unresolved, with some authors advocating more extensive resection as a means of reducing high rates of local recurrence.^29^ Patients with deep-seated, high-grade LPS arising in the extremities should be offered adjuvant or neoadjuvant radiotherapy in line with randomized data for extremity STS.^30^ The addition of perioperative radiotherapy to the management of localized retroperitoneal STS has been reported to produce favorable rates of local recurrence in noncomparative series.^31^ The role of neoadjuvant radiotherapy for high-risk, operable retroperitoneal STS should be defined by the results of the now-completed Phase III Randomized Study of Preoperative Radiotherapy Plus Surgery Versus Surgery Alone for Patients With Retroperitoneal Sarcoma (STRASS) trial (NCT01344018).

Although the role of systemic therapy in early-stage STS remains contentious, the greater chemosensitivity of MLPS may indicate a role for adjuvant chemotherapy in this subtype. A single-arm trial of preoperative trabectedin in locally advanced MLPS showed a pathologic complete response in three of 23 assessable patients and partial radiologic response in seven of 29 evaluable patients.^32^ A recent phase III trial comparing neoadjuvant epirubicin-ifosfamide with subtype-tailored chemotherapy in high-risk, localized STS has suggested similar efficacy between trabectedin in MLPS and the more toxic combination regimen.^33^

Decisions regarding surgical and perioperative management of retroperitoneal LPS should be considered on a case-by-case basis within a specialist multidisciplinary team. Such decisions should balance the specifics of tumor anatomy and subtype biology with aggressive upfront strategies.

### Advanced Disease

The development of unresectable local and/or metastatic LPS is associated with a poor prognosis. Similar to other STS subtypes, standard first-line therapy consists of anthracycline-based schedules.^34^ In the European Organization for Research and Treatment of Cancer (EORTC) 62012 phase III trial, post hoc subgroup analysis indicated no improvement in response rate or overall survival (OS) in patients with LPS treated with combination doxorubicin-ifosfamide compared with doxorubicin alone.^35^ LPS-specific efficacy data are currently unavailable for olaratumab, a PDGFRA-targeting monoclonal antibody recently approved in combination with doxorubicin for untreated advanced STS on the basis of improved OS compared with doxorubicin alone in a randomized phase II trial^36^. Meanwhile, there is clinical evidence that LPS subtypes have differential sensitivity to other available systemic therapies (Table 2).

**Table 2.**
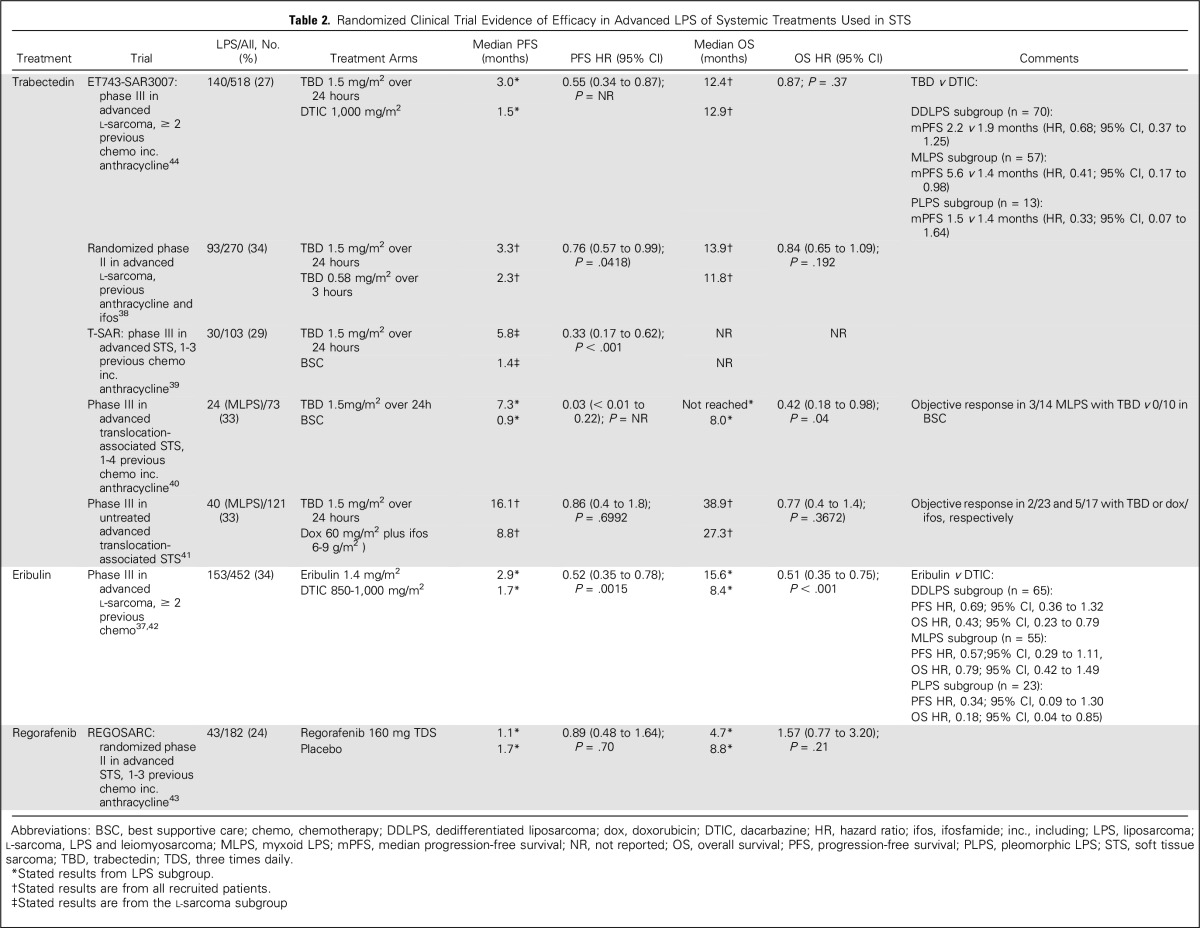
Randomized Clinical Trial Evidence of Efficacy in Advanced LPS of Systemic Treatments Used in STS

Trabectedin is approved for pretreated, advanced LPS on the basis of randomized evidence of improved progression-free survival compared with dacarbazine.^39,44^ Trabectedin is a DNA minor groove binder that also binds to and inhibits the FUS-DDIT3 fusion protein, reversing the blockage of adipocytic differentiation and inhibiting growth in MLPS preclinical models.^45^ This effect is reflected by clinical evidence of pronounced effect of trabectedin against MLPS in randomized studies.^38,40,41^

In a randomized phase III trial of patients with previously treated advanced LPS or leiomyosarcoma, OS was significantly superior in patients who received eribulin, a microtubule inhibitor, compared with standard dacarbazine (HR, 0.768; 95% CI, 0.618 to 0.954; *P* = .017).^42^ Preplanned subgroup analysis indicated that the benefit of eribulin was largely restricted to patients with LPS (HR, 0.51; 95% CI, 0.35 to 0.75; *P* = .006), resulting in approval of the drug in this disease group only.^37^

A single-arm phase II trial of the multitarget tyrosine kinase inhibitor (TKI) pazopanib in advanced STS showed that the drug was inactive in the LPS subgroup, leading to the exclusion of this subtype from the subsequent phase III trial.^46,47^ The differential sensitivity of LPS subtypes to pazopanib has been evaluated in two trials (NCT01506596, NCT01692496). Other multitarget TKIs, including sunitinib, sorafenib, and regorafenib, have shown little indication of efficacy in LPS.^43,48,49^

## INVESTIGATIONAL THERAPIES

Given the frequency and apparent oncogenic role of MDM2 and CDK4 overexpression in WD/DDLPS, there has been significant effort to target these proteins therapeutically.

### MDM2 Antagonists

A number of drugs of different chemical classes have been shown in LPS preclinical models to bind to MDM2 and disrupt its interaction with p53, resulting in upregulation of p53-mediated effects, such as cell cycle arrest and apoptosis.^50-53^ Functional nonmutated p53 seems to be necessary for the anticancer effect of such drugs^51^. In a proof-of-mechanism study of the nutlin-3a-derived drug RG7112 in operable WD/DDLPS, one partial response was seen in 20 treated patients, with best response of stable disease in 14 patients and progression in five.^54^ Comparison of pretreatment and post-treatment tumor samples showed up to a three-fold increase in p53 and p21 expression, with associated increases in tumor apoptosis. Significant neutropenia and thrombocytopenia were among the reported toxicities.

In a first-in-human phase I trial of SAR405838, a spiro-oxinodole inhibitor of MDM2, 49 evaluable patients with LPS were treated.^55^ No objective responses were recorded, but disease stabilization was seen in 32 patients (65%), with an associated 3-month progression-free rate (PFR) of 32%. Thrombocytopenia was the dose-limiting toxicity with a daily dosing schedule, whereas no dose-limiting toxicity was reached with a weekly regimen. In a translational substudy, sequencing of sequential circulating cell-free tumor DNA demonstrated that increases in burden of radiologically apparent disease correlated with increasing levels of peripherally detectable *TP53* mutation.^56^ These data implicate clonal outgrowth of *TP53*-mutated tumor as a potential mechanism of resistance to MDM2 inhibition.

MK-8242 is a small molecule inhibitor of MDM2, with phase I data showing partial responses in three of 42 treated patients with LPS (7%) and stable disease in 31 (74%).^57^ Additional MDM2 inhibitors of several other classes, including DS-3032B, CGM097, and JNJ-26854165, are under ongoing investigation.

### CDK4 Inhibitors

Palbociclib is an inhibitor of CDK4 and CDK6 that is approved for advanced breast cancer and has been shown to induce cell cycle arrest in CDK4-overexpressing LPS cells.^11^ In a single-arm phase II study of palbociclib, 48 patients with WD/DDLPS were treated with a dosage of 200 mg once daily for 14 days every 3 weeks.^58^ *CDK4* was amplified in 92% of patients, whereas pRb expression was intact in 85% of tumors. At this initial dosing level, significant hematologic toxicity, along with one partial response and a 12-week PFR of 66%, was seen. An additional 30 patients with WD/DDLPS were then treated at a lower-dose schedule of 125 mg once per day for 21 days every 4 weeks. In these patients, the 12-week PFR was 57%, with one radiologic complete response and reduced toxicity compared with the original dosing.

Ribociclib is another CDK4/6 inhibitor currently being investigated in WD/DDLPS (NCT03096912). In a phase I trial of ribociclib in LPS, no objective responses were observed (0 of 39 evaluable patients), although disease stabilization surpassing 6 months was seen in six patients (15%).^59^ Toxicities included neutropenia and QTc prolongation.

Other CDK4/6 inhibitors, including the structurally distinct abemaciclib that is able to cross the blood-brain barrier,^60^ are currently under clinical investigation in WD/DDLPS as single agents or in combination with MDM2 or mTOR inhibitors (NCT02846987, NCT02343172, NCT03114527). Preclinical studies have indicated that intact baseline expression of ATRX and demonstrable loss of MDM2 expression after treatment seem to be necessary for CDK4 inhibition to successfully induce cancer cell senescence.^61^ The potential utility of ATRX expression as a biomarker for CDK4/6 inhibitors has yet to be clinically tested.

### Exportin 1 Inhibitors

Exportin 1 (XPO1) is a known mediator of the nuclear export of more than 200 proteins, many of which have tumor suppressor functions. XPO1 overexpression is seen in multiple cancer types, including LPS. Knockdown of XPO1 function in LPS cells induced apoptosis and inhibited tumor growth in LPS xenografts, with treated cells exhibiting upregulation of adipocyte differentiation-related genes and reduced mitosis-related gene expression.^62^ Selinexor, a selective inhibitor of XPO1, was investigated in a phase IA/IB trial in patients with advanced cancer with recently confirmed disease progression, including 19 with DDLPS.^63^ In 15 evaluable patients with DDLPS, no objective responses were seen, but disease stabilization > 4 months was recorded in seven patients (47%), six of whom had a reduction in the target lesion size that did not reach partial response criteria. Recurring grade 3 adverse events included fatigue, anemia, and thrombocytopenia. A placebo-controlled phase II/III trial on DDLPS is now recruiting (NCT02606461).

### PPARγ Agonists

PPARγ is a nuclear receptor that regulates the transcription of genes that are critical for terminal adipocyte differentiation. LPS cells are induced to differentiate in vitro on PPARγ agonist exposure.^64^ Although repurposing PPARγ agonists used to treat diabetes produced disappointing phase II results in LPS,^65,66^ more recent phase I results with efatutazone, a third-generation thiazolidinedione PPARγ agonist, were more encouraging, with partial response sustained at 23 months in one patient with MLPS.^67^ A single-arm phase II trial of efatutazone in advanced MLPS is ongoing (NCT02249949).

### Immunotherapy

In the Sarcoma Alliance for Research Through Collaboration (SARC) 028 phase II study of pembrolizumab in advanced STS, two of 10 patients treated for DDLPS met partial disease response criteria.^68^ Meanwhile, high expression of the cancer testis antigen NY-ESO-1 has been shown in a large proportion of MLPS patients.^69^ Therapeutic targeting of this antigen with peptide vaccine or adoptive cell therapies is currently under way and has been associated with some early encouraging pharmacodynamic and clinical results.^70,71^

### Other Investigational Agents

Sitravatinib (MGCD516) is a TKI active against a broad spectrum of targets, including MET, PDGFRA, c-Kit, and IGF-1R, and is currently under phase II investigation in patients with LPS (NCT02978859). In preclinical work, sitravatinib demonstrated superior antiproliferative effects over other TKIs across a range of STS cell lines and xenografts, including IGF-1R–overexpressing LPS models.^72^ Phase I data showed mucositis, fatigue, and neuropathy as dose-limiting toxicities.

Aurora kinase A (AURKA) is a critical mediator of mitosis that is commonly overexpressed in STS.^11^ Alisertib, an inhibitor of AURKA, exhibits in vitro antiproliferative effects against LPS cells^73^ and was associated with a 73% 12-week progression-free rate in 12 patients with LPS treated within a phase II study of the drug.^74^ There has yet to be additional clinical development of AURKA inhibitors in LPS.

A repurposing phase I/II of the anti-HIV protease inhibitor nelfinavir in LPS showed disappointing clinical efficacy signal despite compelling in vitro data demonstrating the induction of cell cycle arrest and apoptosis in DDLPS cells by the drug via upregulation of SREBP-1, an adipocyte differentiation-related transcription factor.^75,76^

In conclusion, LPS encompasses a number of different subtypes with distinct underlying biology and clinical behavior. Additional work is required to better understand subtype-specific biology and to identify new targets. A recent randomized trial has suggested that neoadjuvant trabectedin may have similar activity to anthracycline and ifosfamide in localized myxoid LPS. Furthermore, two randomized phase III trials have led to the approval of trabectedin and eribulin for advanced pretreated LPS. Despite this, there is a clear need for further development of subtype-specific therapy in LPS. MDM2 and CDK4 inhibitors have shown some evidence of efficacy in WD/DDLPS, but also toxicity. The precise role of these agents remains to be defined, particularly the potential for combination therapy. A number of other promising agents are currently being evaluated in advanced LPS, and the results of these ongoing trials are eagerly awaited. Ongoing national and international collaboration is critical to coordinate research efforts and quickly establish the efficacy of trialed agents while ensuring that high-quality translational research is performed.
